# Shelf Acetabuloplasty in the Treatment of Severe Legg-Calvé-Perthes Disease: Good Outcomes at Midterm Follow-Up

**DOI:** 10.1155/2013/859483

**Published:** 2013-11-27

**Authors:** Andrzej Grzegorzewski, Marek Synder, Krysztof Kmieć, Karol Krajewski, Michał Polguj, Marcin Sibiński

**Affiliations:** ^1^Clinic of Orthopaedics and Paediatric Orthopaedic, Medical University of Łódź, ul. Drewnowska 75, 91-002 Łódź, Poland; ^2^Department of Angiology, Medical University of Łódź, ul. Narutowicza 60, 90-136 Łódź, Poland

## Abstract

The aim of the study was to retrospectively review results of operative treatment for coverage deficit of femoral head in children with severe epiphysis displacement in Legg-Calvé-Perthes (LCP) disease. The material included 23 shelf acetabuloplasty procedures for LCP disease. The average age at diagnosis was 8.1 years (range 4–12). Mean follow-up was 5.8 years (range from 2.2 to 11.2 years). Mean Reimer's index decreased statistically significantly from a mean of 32% before surgery to 10.0% at the last follow-up (*P* < 0.00001). The mean Wiberg center-edge angle increased also statistically significantly from a mean of 17.3° before procedure to 32.3° at the last follow-up (*P* < 0.00001). According to the Stulberg classification, type I was observed in 2, type II in 13, type III in 6, and type IV in 2 hips. There were no differences in the range of motion or leg length discrepancy in preoperative and postoperative standing. Partial, not significant, bone graft resorption was noted in 6 cases in the first 6–9 months after surgery. To conclude, shelf acetabuloplasty allows achieving good midterm results in the treatment of severe stages of LCP disease. The procedure improves coverage of femoral head and allows its remodelling.

## 1. Introduction

More than one hundred years ago the Legg-Calvé-Perthes disease was presented to the world literature. Since that time there are still controversies according the management of this disease. There is no one treatment protocol for children with Legg-Calvé-Perthes (LCP) disease. The primary goal of treatment for Perthes disease is to help the femoral head recover and grow to a normal shape. Treatment aims are to reduce hip pain and stiffness, to reduce hip irritability and restore and maintain hip mobility, and also to prevent the femoral head from extruding or collapsing to regain a spherical femoral head to prevent deformity of the femoral head. In most of the cases the goal of treatment can be achieved by conservative methods; but in severe ones with femoral head deformity and subluxation of the epiphysis the surgery would be indicated. The shelf acetabuloplasty is assumed to stabilize acetabular labrum and it has been proved to have stimulatory effect on acetabular growth [1–4]. It may be performed to assure sufficient coverage of the extruded femoral head due to lack of either concentricity or congruence. This procedure can be performed in the early stage of the disease to prevent further deformation of the femoral head [5] or in the treatment of residual deformity [6].

The aim of the study was to retrospectively review midterm results of operative treatment for coverage deficit of femoral head in children with severe LCP disease.

## 2. Material and Methods

Between 2002 and 2009, a total of 120 children were treated for LCPD in our institution. Vast majority of patients were treated conservatively. From this 120 patients 20 had shelf acetabuloplasty, 3 patients had adductor myotomy, and in other 3 patients a salvage procedure—proximal femoral valgus osteotomy—was performed for residual deformity of femoral head. None of our patients had proximal femoral varus osteotomy or Salter pelvis osteotomy done at this time period.

We retrospectively review results of 20 consecutive patients (17 boys and 3 girls) who underwent 23 shelf acetabuloplasty procedures in our institution for LCP disease. Bilateral procedure was performed in 3 cases. Left hip was treated in 12 and right hip in 11 cases. All those patients were capable of attending final follow-up. The average age of our patient cohort at diagnosis was 8.1 years (range 4–12). Mean age at surgery was 9.3 years (range: 5 to 14 years) and median age 9 years (Table 1). Mean follow-up was 5.8 years (range: 2.2 to 11.2 years) and median follow-up 5.7 years. Before surgery all children had only conservative treatment including physiotherapy, bed rest, and traction in abduction or Petri cast. None of them had surgery on involved hip.

The decision to perform a shelf acetabuloplasty was based on clinical and radiological signs. The indication for surgery on anteroposterior (AP) X-ray film was femoral head subluxation more than 20% according to the Reimer index [7] or the presence of hinge abduction, a flat uncovered femoral head, and the Wiberg center-edge angle [8] less than 20°. Additionally in all cases the lateral shape of acetabulum was classified into group B or C according to Grzegorzewski et al. classification system [1]. Before surgery we tried to restore a good range of motion in the hip joint by using physiotherapy and bed rest and traction in abduction, particularly hip abduction (at least 15°) and internal rotation (at least 10°). These children were all treated after the preferred period of preventive surgery.

Clinical data and radiographs in AP and Lauenstein position were used for evaluation. According to the Herring classification [9] hips were grouped into types B, B/C, and C, and according to the Catterall classification [10] hips were grouped into types II, III, and IV and we looked for “head at risk” signs. The Reinberg classification was used to evaluate the stage of disease (Table 1) [11]. The Reimer index [7], the Wiberg center-edge angle [8], and LLD were evaluated in every case. The Wiberg center-edge angle at the final follow-up was measured to the end of solid bone graft. The leg length discrepancy (LLD) and range of motion in the operated hip joint were also measured. The Stulberg classification was used for final results of radiological assessment [12].

## 3. Shelf Acetabuloplasty Surgical Technique

The reflected tendon of the rectus femoris is divided from the direct portion and dissected posteriorly. A curvilinear slot is produced in the subchondral bone of the anterolateral aspect of the acetabular roof, 3–3.5 cm in length, 2-3 mm in height, and 1 cm in depth, in an ascending direction from lateral to medial and from distal to proximal. One or two cortical grafts are harvested from the iliac wing, tightly inserted in the slot with an extrusion of 1.5–2 cm lying on the hip capsule after its exposure, and secured with the reflected head of the rectus femoris. This is sutured back to its origin on the direct head keeping 5-6 mm of the most lateral aspect of the graft, exposed in such a way as to be in contact with the cancellous and cortical graft harvested from the same iliac wing, and put on top of it. Patient was immobilized in spica cast for 6 weeks after operation and then we started physical therapy and walking with crutches without bearing. Weight bearing was allowed 3-4 months after surgery (depending on the age at time of surgery).

Statistical analysis was undertaken using Statistics for Windows 7.1 Pl. Statistical correlations were performed using the Student's *t*-test and the Pearson correlation test with variance analysis for repetitive measures. A *P* value <0.05 was considered to be significant. 

## 4. Results

Fifteen patients had normal and 3 patients almost normal and painless hip joint mobility at last follow-up. Two patients had limitation of the hip joint range of motion, particularly the abduction and internal rotation (patients with Stulberg type IV). The range of motion has not changed at final follow-up comparing to preoperative status. Three patients (15%) had an obvious Trendelenburg gait, 5 (25%) had a moderate limp, and 12 (60%) had a normal gait pattern. A leg length discrepancy (LLD) before operation was ranging from 1 to 2 cm (average 1.62 cm) and was found in eight patients. At last follow-up LLD was found in 5 patients (range 1-2 cm, mean 1.5 cm).

The results in relation to Harring classification are noted as follows: Harring B—1 patient had Stulberg I, 8 Stulberg II, and 1 Stulberg III; Harring B/C—1 child had Stulberg I, 2 Stulberg II, 3 Stulberg III, and 1 Stulberg IV; Harring C—3 patients had Stulberg II, 2 Stulberg III, and 1 Stulberg IV.

In those children who were 8 years and older, the results are as follows: Harring B—4 patients had Stulberg II and 1 Stulberg III; Harring B/C—2 patients had Stulberg II, 2 Stulberg III, and 1 Stulberg IV; Harring C—2 children had Stulberg II and 2 Stulberg III. 

Femoral head “at risk signs” were present in all cases ranging in number from one to four (one risk sign, 9%; two, 17%; three, 22%; and four, 52%). Mean Reimer's index decreased significantly from a mean 32% before operation to 10.0% at last follow-up (*P* < 0.00001). The mean Wiberg center-edge angle increased significantly from a mean 17.3° before procedure to 32.3° at last follow-up (*P* < 0.00001). According to the Stulberg classification there were type I in 2, type II in 13 (together 15 good results, 65.2%), type III in 6 (satisfactory, 26.1%), and type IV in 2 hip joints (poor, 8.7%). Final radiographic results (Stulberg classes) were not related to gender, involved side, age at diagnosis, number of “head at risk” signs, and Catterall, Herring, and Reinberg stages of the disease (*P* ranging from 0.074 to 0.8).

During operation we did not observe any complications. The lateral part of the acetabulum was intact and there was no cup growth disturbance during follow-up. Nevertheless, during follow-up on X-ray film we detected partial bone graft resorption in lateral part of the graft in 6 cases. The maximum bone disappearing was observed in the first 6–9 months after surgery and did not exceed 7 mm. This phenomenon was seen particularly in poor and satisfactory results according to the Stulberg classification. No patients required additional surgery.

## 5. Discussion

Analysis of results in our patients confirms that shelf acetabuloplasty gives satisfactory results of involved hip. In our opinion it is effective in the management of severely involved hips with subluxation, incongruence, and hinge abduction. We consider shelf acetabuloplasty as an operative treatment in this cases, which allowed for the acetabulum and femoral head restoration when performed in late fragmentation or in early rebuilding stage. The shelf acetabuloplasty provides good containment and gives good support allowing for remodeling of the femoral head. Preventive operative treatment methods for LCPD—proximal femoral varus osteotomy or Salter pelvis osteotomy—are rarely used in our practice.

Wright Perry DC and Bruce reviewed data of 24 patients older than eight years of age who had shelf acetabuloplasty [13]. They noted medial joint space ratio and the acetabular cover ratio improvement. Most of their patients were Stulberg II or III at skeletal maturity [13]. In the study of van der Geest et al. 6 patients at skeletal maturity had good results (Stulberg 1 or 2), 10 hips had a fair result (Stulberg 3), and 2 hips had a poor result (Stulberg 4 or 5), but it was better than the natural history of the disease [14]. These findings are similar to ours. Ghanem et al. reported more satisfactory improvement in clinical and radiological parameters in consecutive series of 30 patients with lateral shelf acetabuloplasty with or without varus osteotomy. At last follow-up all patients were pain free, had satisfactory hip motion, mild or no limping, and most of them were classified as Stulberg 1 or 2 [15].

Hsu et al. performed a systematic review of the medical literature. They found that this procedure significantly improves coverage of femoral head measured by most popular indexes. This is a save procedure and no major complications were noted but does not improve range of motion. However, there is no evidence that the procedure prevents development of degenerative joint disease or improves long-term function [16].

Yoo et al. identified prognostic factors to acetabular remodeling. They concluded that hinge abduction and marked collapse of the epiphysis were negative prognostic factors [17]. However, Freeman et al. reported relatively good results compared with historical controls in 27 children with Perthes disease and hinge abduction at minimum 2 years follow-up [6].

Domzalski et al. found that the shelf acetabuloplasty performed just above lateral rim of cup and joint capsule attachment to the iliac bone stimulates the acetabulum to growth [2].

Some surgeons perform shelf acetabuloplasty at time of diagnosis on all children older than seven or eight years rather than waiting for subluxation that will inevitably occur in that age group [5, 18–20]. Vast majority of the children older than 8 years in the Perthes study group were candidates for surgery because only one patient older than age 8 remained Harring A throughout the course of disease. All others became B, B-C, or C. Children younger than 8 are observed for subluxation and only have surgery when that happens but older children rarely need to wait [19]. Stulberg et al. showed that 100% of children older than 9 will develop poor outcomes unless they are treated [12], but Herring et al. lowered that age to 8 years [19] and Joseph to 7 years [20]. In our study we performed shelf acetabuloplasty on 3 patients younger than 7 years at the time of surgery. We want to emphasize that this procedure was performed for hip incongruence and subluxation after conservative methods of treatment had failed.

In the study of Daly et al. on skeletally mature patients, who were treated with shelf procedure in the initial stage of the disease, 22 of 27 hips were rated as Stulberg groups 1 to 3. Poor results were found mostly in girls over 11 years and with severe form of the disease [18]. Jacobs et al. assessed this procedure in the early stage of LCP disease as appropriate surgical treatment for children older than 5 years of age [5].

There are several complications reported in the literature following this procedure. Growth disturbance of the lateral aspect of the acetabulum did not occur in any of our patients. Similar to Domzalski et al. and Daly et al. [2, 18] we found that acetabulum continues to grow following surgery adequately covering the femoral head. Another reported complication of this procedure is proximal migration of the shelf.

 When bony shelf is stabilized using the reflected head of the rectus femoris tendon, proximal shelf migration is unlikely. We observed bone graft resorption in lateral part of the graft in our study group. In our opinion the reason of bone disappearing is most likely not loading in this area of the acetabulum.

This paper has limitations. The most important is relatively small number of cases, which makes statistical analysis not reliable.

## 6. Conclusions

Shelf acetabuloplasty allows achieving good midterm outcomes in the treatment of severe LCP disease. The procedure improves coverage of femoral head allowing its remodelling.

## Figures and Tables

**Table 1:** Selected epidemiological and radiographic data on 20 patients treated with shelf procedure for Perthes disease.

No.	Age (years)	Gender	Side	Before operation					At the final follow-up		
				Reimer's index	Wiberg angle	Reinberg	Catterall	Herring	Reimer's index	Wiberg angle	Stulberg
1	9	♂	Right	37%	17°	IV	III	B	5%	40°	II
2	10	♂	Right	32%	15°	III	III	B/C	0%	34°	I
3	6	♂	Right	29%	18°	III	III	B	0%	40°	I
4	10	♂	Left	32%	16°	IV	IV	C	4%	38°	II
5	9	♂	Right	30%	17°	III	III	B/C	7%	40°	III
6	9	♂	Left	25%	19°	III	III	B	4%	40°	II
7	12	♂	Left	31%	18°	III	III	B/C	9%	33°	III
8	11	♂	Right	41%	14°	III	III	B	2%	34°	III
9	5	♂	Left	27%	19°	IV	IV	C	5%	34°	II
10	12	♂	Left	36%	16°	IV	IV	C	23%	25°	III
11	7	♂	Left	37%	17°	II	III	B/C	21%	26°	III
12	7	♀	Right	23%	19°	III	III	B	2%	36°	II
13	13	♂	Right	30%	18°	III	IV	C	2%	36°	II
14	6	♂	Left	35%	18°	II	II	B	11%	32°	II
15	14	♂	Right	43%	14°	IV	III	B	19%	28°	II
16	9	♂	Left	33%	18°	III	III	B/C	9%	33°	II
17	14	♀	Right	31%	17°	IV	III	B/C	7%	31°	II
18	7	♂	Left	30%	19°	II	II	B	13%	33°	II
19	10	♀	Right	35%	16°	III	III	B	20%	33°	II
20	12	♂	Right	26%	19°	III	III	B/C	16%	23°	IV
21	7	♂	Left	30%	18°	II	III	B	18%	26°	II
22	8	♂	Left	26%	19°	III	IV	C	14%	23°	IV
23	10	♂	Left	35%	17°	III	IV	C	21%	25°	III
